# Protein restricted diet during gestation and/or lactation in mice affects ^15^N natural isotopic abundance of organs in the offspring: Effect of diet ^15^N content and growth

**DOI:** 10.1371/journal.pone.0205271

**Published:** 2018-10-10

**Authors:** Karine Bernardo, Céline Jousse, Pierre Fafournoux, Anne-Marie Schiphorst, Mathilde Grand, Richard J. Robins, Régis Hankard, Arnaud De Luca

**Affiliations:** 1 Inserm UMR 1069, Tours, France; 2 University Hospital of Tours, Tours, France; 3 F Rabelais University, Tours, France; 4 Clermont Auvergne University, INRA, UNH, Unité de Nutrition Humaine, Clermont-Ferrand, France; 5 Elucidation of Biosynthesis by Isotopic Spectrometry Group, CEISAM, CNRS-University of Nantes, UMR 6230, Nantes, France; Charles P. Darby Children’s Research Institute, 173 Ashley Avenue, Charleston, SC 29425, USA, UNITED STATES

## Abstract

**Objectives and study:**

This study aimed at measuring the effect in normal to restricted protein diets with specific ^15^N natural isotopic abundance (NIA) given during gestation and/or lactation on the ^15^N NIA of fur, liver and muscle in dams and their offspring from birth to adulthood. The secondary aim was to study the effect of growth on the same parameters.

**Methods:**

Female Balb/c mice were fed normal protein diet containing 22% protein or isocaloric low protein diet containing 10% protein throughout gestation. Dam’s diets were either maintained or switched to the other diet until weaning at 30 days. All animals were fed standard chow thereafter. Offspring were sacrificed at 1, 11, 30, 60, 480 days and a group of dams at d1. Growth was modeled as an exponential function on the group followed up until 480 days. Fur, liver and muscle were sampled at sacrifice and analyzed for bulk ^15^N NIA. Fixed effects and interactions between fixed effects and random elements were tested by three-way ANOVA.

**Results:**

Higher ^15^N NIA in the diet resulted in higher organ ^15^N NIA. Switching from one diet to another changed ^15^N NIA in each organ. Although dam and offspring shared the same isotopic environment during gestation, ^15^N NIA at day 1 was higher in dams. Growth rate did not differ between groups after 10 days and decreased between 1 and 5 months. ^15^N NIA differed between organs and was affected by growth and gestation/lactation.

**Conclusion:**

Dietary ^15^N NIA is a major determinant of the ^15^N NIA of organs. ^15^N NIA depended on organ and age (i.e. growth) suggesting an effect of metabolism and/or dilution space. Post-natal normal-protein diet of lactating dams could reverse the effect of a protein-restricted diet during gestation on the offspring growth. Measuring ^15^N NIA in various matrices may open a field of application particularly useful in studying the pre- and post-natal origins of health and disease.

## Introduction

^15^N is a stable isotope of nitrogen present in nature, accounting for 0.4% of N in whole body pools [1]. ^15^N natural isotopic abundance (NIA) in body proteins is determined by the ^15^N values of nutrient and by N metabolism [2].

NIA values (δ^15^N) have been shown to be a valuable index of nutrient intake and metabolism in animals [3,4]. The observed enrichment in animal’s body proteins compared to their diet consists on the trophic effect, resulting in the cumulative isotopic effects associated in metabolic pathways [5]. Furthermore, a discrimination exists against heavier isotope (^15^N) in urea formation, which contributes to the trophic effect [6]. Poupin et al. provide robust arguments for the variation in ^15^N NIA according to protein metabolism in rats [4,7]. They modeled body compartments involved in protein metabolism and observed that ^15^N NIA depended on protein turnover. ^15^N NIAs in tissues differed according to the protein turnover of each organ, e.g. the liver had a higher ^15^N NIA than the muscle [5]. The intensity of the isotopic shift seems to depend on the liver metabolic pathways and their activity, i.e. protein turnover, whether modulated by dietary intake or pathological state [6,8]. Fractionation may then vary with fluxes and protein synthesis or degradation. Variations in fluxes could result in a compartment change, which could modify other compartments by subsequent fluxes. During fluxes stabilization, even if the stable state is different from the previous one, the fractionation could return to the previous level, making this marker dynamic [9].

Epidemiological studies carried out in humans show that intrauterine environment, especially maternal nutrition, play an important role in the appearance of metabolic diseases in adulthood. This is the concept of fetal programming, i.e. Developmental Origins of Health and Disease (DOHaD) [10]. Protein restriction in mice during gestation and lactation induced in offspring a hypermetabolic phenotype in adulthood with lower weight, less fat mass and increased food intake, associated with altered methylation of the promoter region of the leptin gene [11]. This suggests a metabolic impact of protein restriction during early life.

More than being a biomarker of food isotopic composition, ^15^N NIA measurement in organs may provide a marker to assess the impact of early diet composition on protein metabolism and growth, arguing for fetal programming. This study aimed at measuring the effect in normal to restricted protein diets with specific ^15^N NIA given to female mice during gestation and/or lactation on the ^15^N NIA of fur, liver and muscle in dams and their offspring from birth to adulthood. The secondary aim was to study the effect of growth on the same parameters.

## Material and methods

### Study design

#### Animal protocols

Balb/c mice (obtained from JANVIER Labs Saint Berthevin 53941 France) were used. All animals were housed in plastic cages and subjected to a 12-h light-dark cycle at a temperature of 22 ± 2°C. All animals had *ad libitum* access to food and water at all times, unless indicated otherwise.

One hundred and forty virgin female Balb/c mice (F0) aged between 2.5 and 4 months old were used. Pairs of female mice were mated with a single male and were allocated into 2 groups fed either a normal protein diet (NPD) containing 22% protein or an isocaloric low protein diet (LPD) containing 10% protein throughout gestation. After delivery, female mice were once again randomized between diet LPD or NPD during lactation. Finally, 4 groups were formed: NPD during gestation and lactation or NPD only during gestation then LPD during lactation and the same thing for LPD. Seven female mice who ate pups were excluded from analyses.

Only litters of 4–11 pups (F1) were included in subsequent experiments. Only males were analyzed to avoid any influence of the female hormonal system on metabolism. After weaning at 30 days of age, the male offspring were single-housed and fed a standard chow diet (A03; Safe, Augy, France) *ad libitum* throughout life. The diets compositions are given in Table 1.

**Table 1:** Diet compositions.

	NPD	LPD
**Total protein (%)**	**22**	10
**A03 (%)**	45	45
**Colza oil (%)**	2	2
**Sunflower oil (%)**	0.4	0.4
**Groundnut oil (%)**	1.6	1.6
**Vitamins mix (%)**	0.55	0.55
**Mineral mix (%)**	3.85	3.85
**Sucrose (%)**	8.25	8.25
**Agar-agar (%)**	1.65	1.65
**Casein (%)**	**12**	0
**Starch (%)**	**24.7**	36.7

Differences in bold; A03 contained: cereals 69.2%, animal proteins (fish) 6%, and vegetable proteins 20.2%. The vitamin mixture used is UAR200 (Safe) and the mineral mixture is UAR205b (Safe) 4.6%. LPD: low protein diet (10% protein, isocaloric), NPD: normal protein diet (22% protein).

Growth study was performed on a separate set of animals with the same experimental procedure as above. In fact, animals had to stay alive until the end of observation i.e. 17 time points from day 10 to month 16. Four animals were followed-up in group NPD/NPD, 6 in group LPD/LPD, 8 in group NPD/LPD and 7 in group LPD/NPD.

#### Experimental procedures

**Fig 1:**
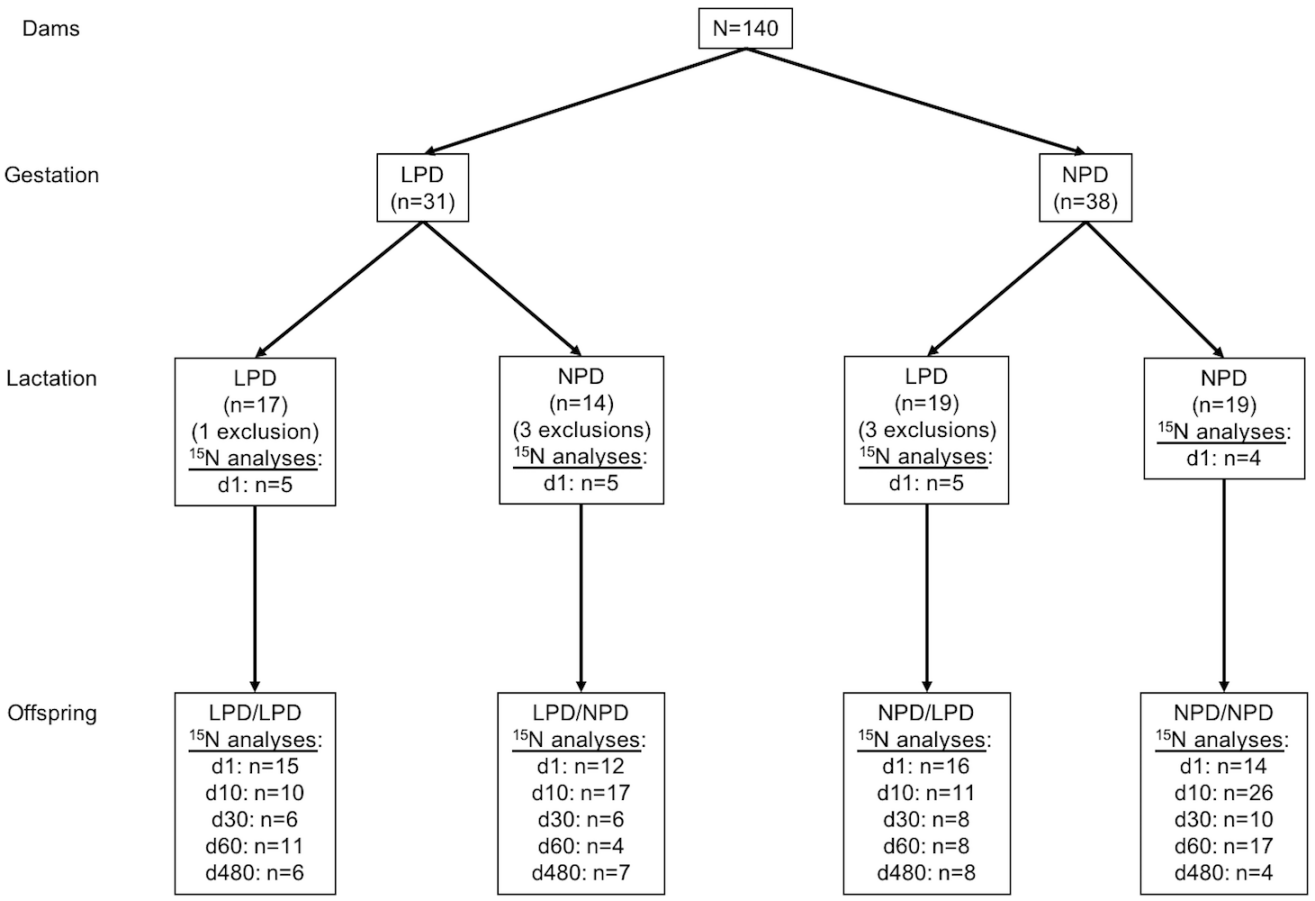
Experimental diagram. Exclusion reason was eating pups. Diet groups: gestation/lactation. LPD: low protein diet (10% protein, isocaloric), NPD: normal protein diet (22% protein), d: day.

**Sample collection and preparation** Body weights were monitored at various ages throughout life for both F1 male mouse groups. Male mice of each group were sacrificed at 1, 11, 30 60, and 480 days old (designated d1, d11, d30, d60 and d480, respectively) and samples taken of fur, liver and leg muscle gastrocnemius for analyses (Fig 1). Dams were sacrificed at d1 and same samples were taken. One and 11-day-old mice were killed by decapitation and 1 to 16-month-old mice were sacrificed by cervical dislocation under KX anesthesia (ketamine/xylazine solution in saline; i.p.). Liver and muscle samples were frozen (-20° C) and lyophilized (HETO tower Dry LL1500 Freeze Dryer, Thermo Fisher Scientific, Bremen, Germany), fur samples were transferred to a small glass bottle and washed in cyclohexane (2 x 2 mL, 30 min) to remove sebum (lipids) and residues. Residual traces of solvent were removed by evaporation at 45° C under a stream of pure nitrogen gas. The sample was cut into small sections (1 mm or less).

An aliquot of each sample (~0.7 mg for fur, ~1 mg for liver, ~0.8 mg for muscle, giving ~0.08 mg N) was weighed with 10^−6^ g precision (ultra-microbalance XP6U, Mettler Toledo, Columbus, OH, USA) into tin capsules (solids “light” 4 x 6 mm, Thermo Fisher Scientific, Bremen, Germany). All samples were analyzed in triplicate and considered valid if the standard deviation between the values was less than 0.3‰.

To obtain the ^15^N NIA of the different diets, the pellets were broken and washed extensively with cyclohexane to remove lipids. The residual solid was dried and ground to a fine homogeneous powder. The measurements were carried out with 5 analyses per diet.

### Isotopic analysis

The ^15^N/^14^N (δ^15^N) isotope ratio was obtained by isotope ratio measurement by mass spectrometry (irm-MS) using a Sigma2 spectrometer (Sercon Instruments, Crewe, UK, http://www.sercongroup.com) linked to a Sercon elemental analyzer as described previously [12]. Isotope ratio δ^15^N (‰) was expressed relative to the international reference using the equation:

$$\delta (‰)=(\frac{R}{Rstd}-1)\text{x}1000$$

where *R* is the isotope ratio of the sample and *Rstd* the isotope ratio of the reference: atmospheric N_2_.

### Statistical analysis

The effects of time (d11, d30), gestation and lactation diets on ^15^N NIA in fur, liver, muscle and weight were tested for main effects and interaction using 3-way ANOVA. Post-hoc tests were performed using Tukey test for more than two groups.

The effect of diet during gestation groups in dams (F0) and offspring (F1) liver ^15^N NIA at d1 was tested using a one factor ANOVA.

In order to take into account the shape of growth curves two models were tested: 1) a three factors exponential model: weight = a + b•e^(c^•^t)^ with a = asymptote, b = scale and c = growth rate, and t = time and 2) a four factors bi-exponential model: weight = a •e^(-b^•^t)^ + c •e^(-d^•^t)^ with a = scale 1, b = decay rate 1, c = scale 2, d = decay rate 2 and t = time. Best model was that with the highest r^2^. The first derivative was calculated at each time point for individuals and compared using two-way ANOVA for group, time and interaction.

Data were expressed as means ± standard error of the mean (SEM). *P* values <0.05 were considered statistically significant. All statistical analyses were computed using JMP 10.0.2 software (SAS Institute Inc. Cary. NC. USA).

### Ethics

Investigations were conducted according to the guiding principles for the use and care of laboratory animals and in compliance with French and European regulations on animal welfare (Décret 2001–464, 29 May 2001 and Directive 2010/63/EU, respectively). The project as a whole was approved by the Animal Experimentation Ethics Committee for Auvergne region (France) C2EA-02 under the approval number 9556–2016122214418591.

## Results

### ^15^N NIA of mice chows

^15^N NIA was higher (p<10^−4^) in NPD (3.78±0.14‰) than in LPD (2.59±0.14‰) and A03 (2.65±0.14‰) diets, with no difference between the LPD and A03 diets.

### Natural isotopic abundances in organs

**Fig 2:**
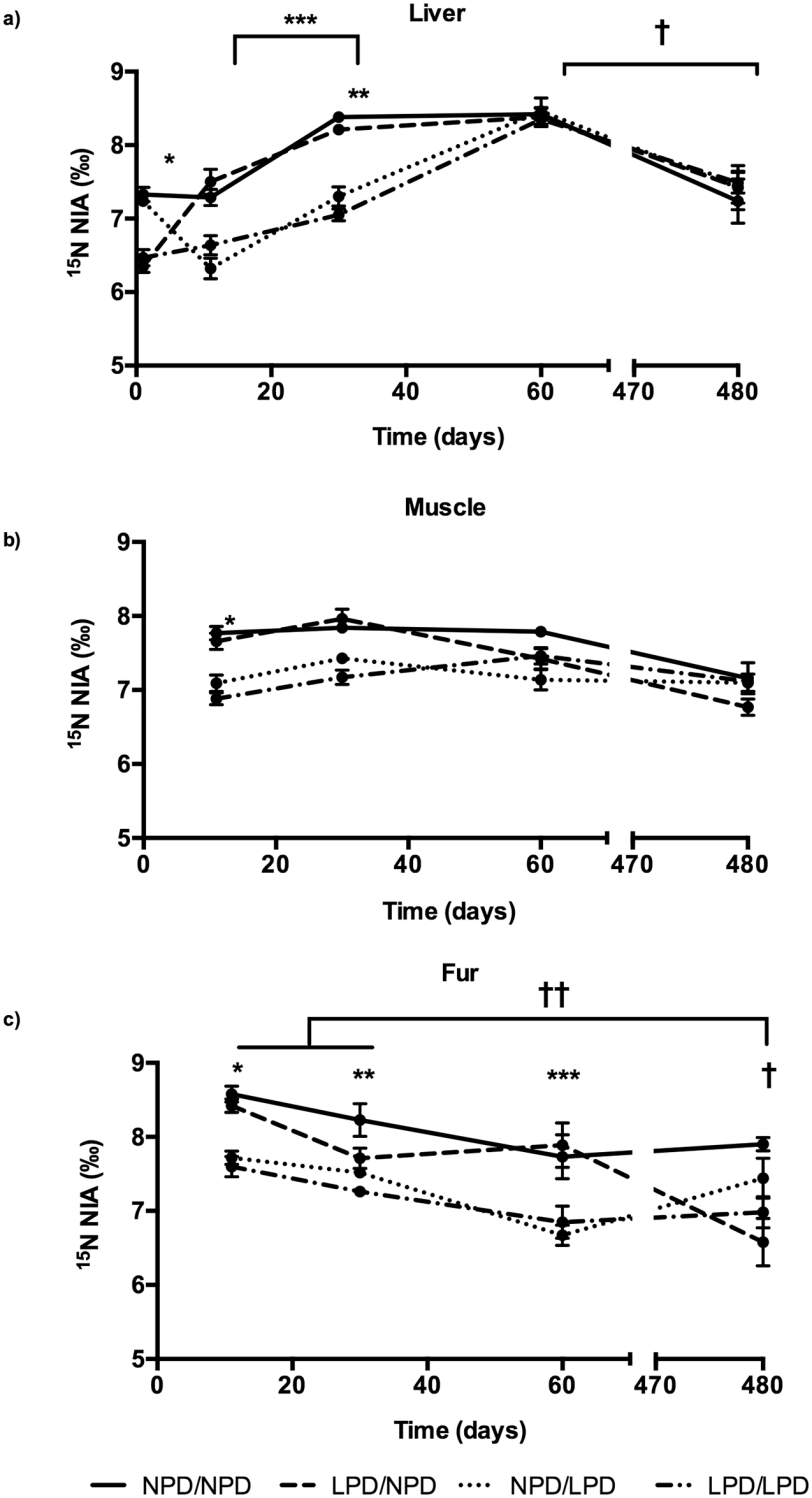
^15^N NIA in organs. a) Liver; b) Muscle; c) Fur. Significant effect of time (p<10^−4^) and group (p<10^−4^) with significant interaction between the two factors (p<10^−4^) on ^15^N NIA in liver, muscle and fur. a) * P<0.05 between NPD in gestation and LPD in gestation, ** P<0.05 ^15^N NIA higher in the NPD/NPD and LPD/NPD groups than in the LPD/LPD and NPD/LPD groups, *** P<0.05 In all groups, d30 ^15^N NIA increased up to d60 † and decreased afterwards (d480). b) *P<0.05 between both NPD/NPD—LPD/NPD groups and LPD/LPD—NPD/LPD groups. c) *P<0.05 between both NPD/NPD—LPD/NPD groups and LPD/LPD—NPD/LPD groups. **P<0.05 between NPD/NPD group and the other three diet, ***P<0.05 NPD/NPD and LPD/NPD groups had higher ^15^N NIA values, † P<0.05 between the LPD/NPD and the NPD/NPD groups, †† P<0.05 between d11-d30 and d480 in the LPD/NPD group. Diet groups: gestation/lactation. LPD: low protein diet (10% protein, isocaloric), NPD: normal protein diet (22% protein). NPD/NPD group: d1: n = 14, d11: n = 26, d30: n = 10, d60: n = 17, d480: n = 4; LPD/NPD group: d1: n = 12, d11: n = 17, d30: n = 6, d60: n = 4, d480: n = 7; LPD/LPD group: d1: n = 15, d11: n = 10, d30: n = 6, d60: n = 11, d480: n = 6; and NPD/LPD group: d1: n = 16, d11: n = 11, d30: n = 8, d60: n = 8, d480: n = 8.

We observed a significant effect of time (p<10^−4^) and group (p<10^−4^) with significant interaction between the two factors (p<10^−4^) on ^15^N NIA in all organs (Fig 2A, 2B and 2C). S1 Table and S1 Data are available as supplementary data.

#### ^15^N NIA in liver

At d1, ^15^N NIA in the liver was higher in the offspring whose dams were fed NPD during gestation (NPD/NPD and NPD/LPD groups) than in those fed LPD (Fig 2A). In young mice whose dams were LPD fed during gestation and NPD fed during lactation (LPD/NPD), the ^15^N NIA values rose during the first 11 days to a comparable value to that in the former two groups, while for the NPD/LPD group the opposite trend was observed. These relationships were still observed at the end of lactation (d30): ^15^N NIA in liver was higher in the NPD/NPD and LPD/NPD groups than in the LPD/LPD and NPD/LPD groups. During the following 30 days, with all animals consuming the A03 diet, this difference disappeared, the values for the LPD mice rising to the same as those for the NPD mice by d60. Thereafter, all animals showed an indistinguishable downward trend by d480.

In all groups, weaning (d30) ^15^N NIA increased up to d60 and decreased afterwards (d480). We did not observe any difference between groups once mice chow was identical (d60 and d480).

#### ^15^N NIA in muscle

Because dissection was too difficult in animals weighing around 1g, samples could not be obtained at d1. However, in view of the relationship with diet observed in the liver (Fig 2B), it is likely that the same relationship existed in 1-day-old young, with young from NPD-fed dams having ^15^N NIA values greater than the LPD-fed group. At d11, ^15^N NIA values in muscle was higher in both NPD/NPD and LPD/NPD groups than in the LPD/LPD and NPD/LPD groups (Fig 2B), as seen for liver (Fig 2A). By d60, following 20 days with all animals consuming the A03 diet, no differences were observed between groups, a trend that persisted at d480.

We did not observe any difference between any time points within a same group.

#### ^15^N NIA in fur

Because mice at birth are nude, data cannot be provided at d1. At d11, ^15^N NIA in fur was higher in the NPD/NPD and LPD/NPD groups than in the LPD/LPD and NPD/LPD groups (Fig 2C), essentially as observed for muscle and liver. In contrast, however, by d30 only the NPD/NPD group was distinguishable from the other three diets, although at d60 both the NPD/NPD and the LPD/NPD had higher ^15^N NIA values. At d480, ^15^N NIA values in fur was lower in the LPD/NPD than in the NPD/NPD groups.

We did not observe any difference with time for the LPD/LPD, NPD/LPD, NPD/NPD groups. In the LPD/NPD group, the ^15^N NIA value at d480 was lower than that at d11 and d30.

#### Inter-organs ^15^N NIA over time in F1

**Fig 3:**
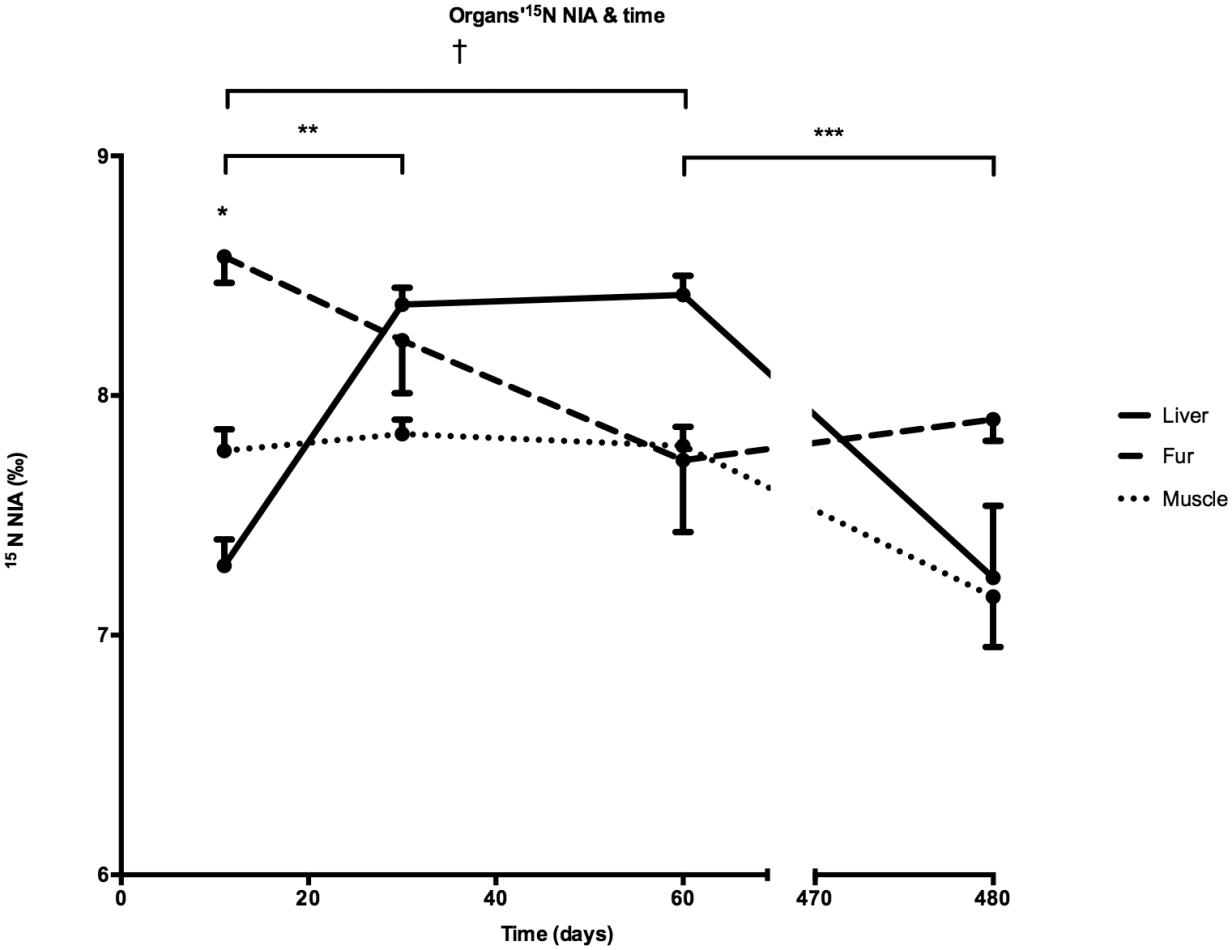
Inter-organs ^15^N NIA over time in F1 mice. * P<0.05 between the fur and both the muscle and the liver, ** P<0.05 between ^15^N NIA in the liver at d11 and d30, *** P<0.05, and decreased from d60 to d480, † P<0.05 between ^15^N NIA in the fur at d11 and d60. Number of samples in each tissue (Muscle, Fur and Liver): d1: n = 57, d11: n = 64, d30: n = 30, d60: n = 40, d480: n = 25.

At d11, ^15^N NIA in the fur was higher than in the muscle and in the liver. No differences between organs were observed at any other time points (Fig 3 and S2 Table as supplementary data).

^15^N NIA increased in the liver from d11 to d30, remained stable until d60 and decreased from d60 to d480 (Fig 3 and S2 Table). In the fur, ^15^N NIA at d11 was higher than at d60 with no differences between any other time points. No differences between time points were observed for muscle.

### Growth

Weight was measured at sacrifice only in mice at d1, d11, d30 and d60 (Table 2). Although there was a trend toward a higher weight at d1 in mice whose dams were fed NPD during gestation it did not reach significance: 1.70±0.03 g (N = 30) for NPD vs. 1.66±0.03 g (N = 26) for LPD. Power analysis set the number of individuals to 294 to reach significance.

**Table 2:** Weight of mice at different time-points.

Diet group	Days				
	1	11	30	60	480
**NPD/NPD**	1.69±0.04a n = 14	7.04±0.16b n = 47	16.3±0.39d n = 24	24.3±0.36f n = 17	34.4±0.64h n = 4
**LPD/NPD**	1.52±0.05a n = 11	7.24±0.12b n = 27	16.4±0.39d n = 10	23.2±0.88fg n = 4	33.4±0.53h n = 7
**NPD/LPD**	1.71±0.03a n = 16	5.57±0.16c n = 23	13.8±0.43e n = 15	23.1±0.39fg n = 7	30.5±0.62i n = 8
**LPD/LPD**	1.76±0.03a n = 15	5.03±0.14c n = 27	12.6±0.27e n = 17	21.2±0.32g n = 11	30.1±0.93i n = 6

Results are Mean±SEM; values with the same letter are not statistically different (multi-way ANOVA and Tukey post-hoc test, significance level, p<0.05). Diet groups: gestation/lactation. LPD: low protein diet (10% protein, isocaloric), NPD: normal protein diet (22% protein).

Weights of NPD/NPD mice were higher than of LPD/LPD mice at all subsequent time points. When NPD gestational diet was switched after birth to LPD, the weight gain from d11 to d480 was indistinguishable from animals fed LPD/LPD. Similarly, when LPD gestational diet was switched after birth to NPD diet, the weight gain was equivalent to that of animals fed NPD/NPD from d11 to d480.

**Fig 4:**
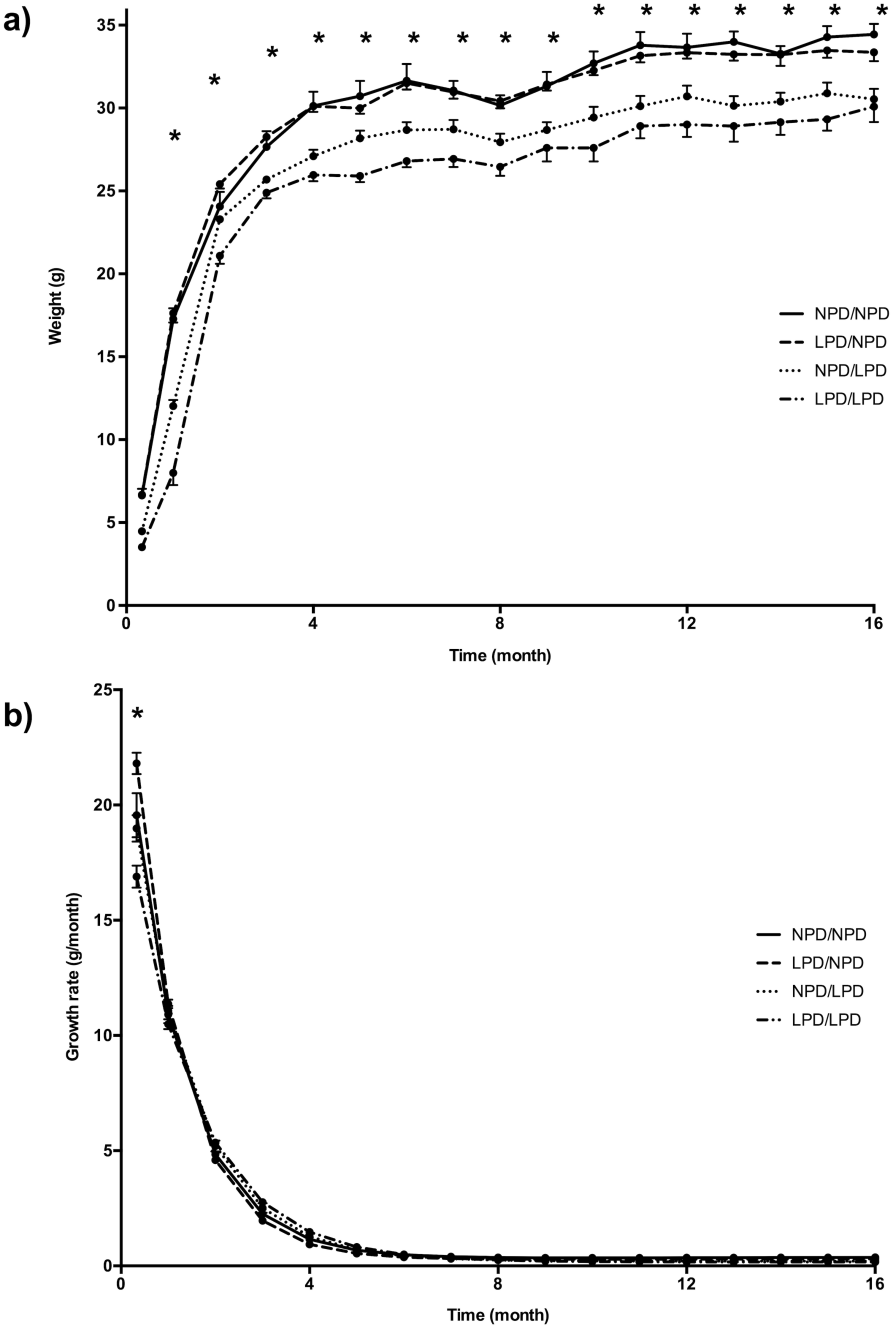
Growth in the four groups of mice. a) Weight charts in the 4 groups of mice; b) Growth rate. a) * P<0.05 between NPD/NPD mice and LPD/LPD mice. Graphically, post-natal nutrition seems more predictive of growth than pre-natal nutrition. b) growth rate at d10 was lower in LPD/LPD mice and higher in LPD/NPD mice than in any other group. Diet groups: gestation/lactation. LPD: low protein diet (10% protein, isocaloric), NPD: normal protein diet (22% protein). The number of animals is available in Table 2.

Weight was recorded monthly on the same mouse and for each group only in mice followed up until d480 (Fig 4A). From this, growth was modeled (Table 3). Four-factor bi-exponential model showed higher r^2^ (0.964 vs. 0.955) and was chosen for calculation of first derivative i.e. weight gain for each mouse and time point (Fig 4B).

**Table 3:** Growth modelling.

	Scale 1	Decay 1	Scale 2	Decay 2
**NPD/NPD**	-29.176±1.193	0.850±0.077	29.197±0.619	-0.011±0.002
**LPD/NPD**	29.357±0.445	-0.009±0.001	-30.240±0.962	0.939±0.064
**NPD/LPD**	27.835±0.517	-0.007±0.002	-30.511±0.861	0.774±0.050
**LPD/LPD**	25.991±0.758	-0.006±0.002	-29.866±1.089	0.691±0.061

Weight over time modeled using a four-factors bi exponential model: weight = a •e^(-b^•^t)^ + c •e^(-d^•^t)^ with a = scale 1, b = decay rate 1, c = scale 2, d = decay rate 2 and t = time. Results are means±SEM. Diet groups: gestation/lactation. LPD: low protein diet (10% protein, isocaloric), NPD: normal protein diet (22% protein). All factors differed between groups. Decay 2 factor differed between ND and all other groups.

Three-factor ANOVA showed a significant effect of lactation diet and time on weight and weight gain without any effect of gestation diet (p<10^−4^).

Weight gain at d10 was lower in LPD/LPD mice (16.9±0.48 g.month^-1^) than in any other group, respectively for NPD/LPD, NPD/NPD and LPD/NPD: 18.98±0.40, 19.56±0.96, and 21.80±0.56 g.month^-1^. Weight gain of LPD/NPD was higher than in any other group. Weight gain was similar in all groups from d30 to d480 (Fig 4B).

#### Effect of gestational diet on dams (F0) and offspring (F1) at d1

At d1, liver ^15^N NIA was higher in dams than in the offspring at d1 (P<10^−4^). Liver ^15^N NIA was higher in dam and offspring fed NPD than in those fed LPD (P<10^−4^) (Table 4). The difference F0-F1 in liver ^15^N NIA at d1 was not different between diet groups (P = 0.15).

**Table 4:** ^15^N NIA in the liver in F0 and F1 animals of the same family.

	F0	F1
**NPD**	8.35±0.07	7.30±0.09
**LPD**	7.27±0.05	6.46±0.12

Results are Mean±SEM; LPD: low protein diet (10% protein, isocaloric), NPD: normal protein diet (22% protein). LPD dyads: n = 10, NPD dyads: n = 9.

## Discussion

The ^15^N NIA values of organs were determined by the ^15^N NIA values of the diet over both the pre- and post-natal period. It differed between organs for a given diet and followed dietary changes. For the same diet, ^15^N NIA changed with time during the first sixty days where growth was present. Post-natal normal protein diet of lactating dams could reverse the effect of a protein restricted diet during gestation on the growth of offspring.

### ^15^N NIA of organs depends on dietary ^15^N NIA

^15^N NIA was higher in NPD chow because it was made of LPD chow with added casein. Casein had a higher ^15^N NIA than LPD and A03 (usual mice chow) diets, both based on the same protein source.

Whether given to the dam during gestation, or postnatally during lactation, a diet with a higher ^15^N NIA value increased ^15^N NIA of organs. Moreover, switching diet from LPD to NPD or vice versa, i.e. providing a switch in dietary ^15^N NIA, induced a concomitant shift in the ^15^N NIA of the organ. This was most evident in the liver for which we have data at d1. This suggest that ^15^N NIA of dams’ diets directly affected the ^15^N NIA of organs in the offspring. After weaning, differences between groups of ^15^N NIA in the liver, the organ with the fastest protein turnover rate [13], tended to fade away since the ^15^N NIA in the diet was identical.

### ^15^N NIA values differed between organs

For a given diet ^15^N NIA values differed between organs. At d11, ^15^N NIA was higher in the fur than in the muscle and liver. This ranking changed with the age of the animals since ^15^N NIA in the liver became higher than in the fur at d60. This suggests that organ specific metabolic characteristics and/or compartmental distribution is another determinant of organ ^15^N NIA. The faster protein turnover rate in the liver [13] probably explains why, in the present study, it is the organ where changes in the ^15^N NIA in the diet had the greatest impact on organ^15^N NIA values. In contrast, in muscle the slower protein turnover rate might be expected to have blunted the effect of diet on organ ^15^N NIA. The fur is a special case, since there is essentially no turnover (bar hair loss) so that keratin is not reincorporated into the whole-body turnover rate. In this tissue, ^15^N NIA acts like a “tape recorder” and ^15^N NIA measured on the whole fur integrates the effect of metabolism and environment over the whole time-period during which the hair has grown. In human research, this provides a helpful index of the impact of metabolic and environmental changes on hair keratin synthesis. Some groups have developed extensive research on isotopic compartmental shifts in the rat [9].

### Effect of diets on growth rate

Animals fed NPD during gestation and lactation or whose diet was switched from LPD to NPD during lactation had a higher adult weight. Since weight was identical between groups at birth, different at d11, but with equal growth rate thereafter, differential growth rate must have occurred within the first ten days. The difference of birthweight was weak and needed a larger number of animals to reach statistical significance, so significant intrauterine growth retardation could not be shown. The apparent graphical inconsistency of weight between groups at d1 (LPD/NPD and LPD/NPD groups) is partly due to the imprecision of birth hours since weight at d1 was only measured in the morning. However, the present model provided restricted nitrogen intake and not a nitrogen free diet. Nevertheless, the present data suggest that post-natal nitrogen nutrition might have the principal effect on growth, since a change in the protein content could reverse the negative impact of low protein diet during gestation on final weight and birthweight. We failed to bring out an effect of gestational diet on post-natal weight and weight gain using a 3-factor ANOVA (time, gestational diet, lactation diet). This result must be taken with caution since based on the population we used for growth modeling therefore missing any weight change before d10. The animals in the NIA experiment show in fact a crossing when mother diet changed from LPD to NPD or conversely. More time points during this period would have been necessary to show more precisely such an effect. Considering the different life spans between humans and mice, this period fits with the so-called 1000 days, a susceptibility window to “program” health status at the adult age in humans. Environment changes during this period may have harmful effects, such as lowering the final weight.

### The ^15^N NIA of organs changed with age and between dams and offspring

The ^15^N NIA of F1 organs increased from d1 to d60 in the liver and decreased over the same period in the fur. This is even noticeable in animals fed the same diet from d1 to d30. Plotting weight over time shows that growth occurred until the “adult” weight is reached between d60 and month 4. This suggests that growth-associated metabolic changes had a specific effect on the ^15^N NIA of organs. Similarly, dam and offspring ^15^N NIA values in the liver differed, even though they shared the same exogenous source of ^15^N; that of the dam’s diet. We did not find a different shift between F0 and F1 between diet groups, suggesting that the diet-induced alteration of protein metabolism impacts the fetus. This result goes against the common belief is that fetal metabolism is the priority. In a previous work, we observed a difference in humans between the mother and her child [14], where ^15^N NIA in hair was higher in F1 (child) than in the F0 generation (mother) by an average of 1‰. The relation between F0 and F1 was in the opposite way compared to the present study but the conditions were different i.e. a normal nutritional environment and furthermore the material was different, with different kinetics, since liver is a high-level metabolic rate organ and hair is a terminal material with no turnover rate. Altogether, these observations suggest a differential effect of fetal and adult metabolisms on the ^15^N NIA of organs.

### Limitations

The main limitations of our study were the difference of ^15^N NIA in diets and the impossibility to analyze ^15^N NIA in mice milk. It was technically difficult to obtain similar ^15^N NIAs and same proteins in the three diets, which results in a change of the isotopic environment of the animals two or three times in a very short period of time. This point hardens the interpretation of the results. ^15^N NIA in mice milk could have allowed measuring directly the ^15^N NIA of what the offspring ingested but the quantity of milk was not sufficient for analysis.

This study was not designed to test the effect of hormones on ^15^N NIA that is seldom documented in the literature. The present study is based on that protein restricted diet induced changes in DNA methylation and expression of leptin gene [11]. Food-intake behavior and body composition were altered consequently. Effect on other hormones such as oxytocin, prolactin or insulin but also amino acids need to be studied specifically [15,16].

### Perspectives for research in nutrition and metabolism

An effect of protein restricted diet during gestation/lactation on leptin expression and response to meal has previously been reported [11]. However, the effect of diet on protein metabolism in dams and their offspring could not be ruled out in the absence of tracer methodology. We have taken advantage of ^15^N NIA values in diet to test whether ^15^N NIA in organs differed between groups. The present study provides evidence that organ ^15^N NIA is not solely driven by ^15^N NIA in the diet. Within the same group and organ ^15^N NIA varied with time, suggesting a growth-associated metabolic effect. ^15^N NIA differed between organs as a possible consequence of organ-specific protein turnover rate and/or compartmental fluxes.

In humans, ^15^N NIA measurements can help to discriminate healthy individuals [17,18] from those displaying severe pathologies affecting nutrition, including anorexia nervosa [19], liver diseases [20] and diabetes nephropathy [21]. Hair at birth is a protein matrix formed during the fetal period that may provide a non-invasive marker, particularly useful in pediatrics at birth and during early growth. Epidemiological studies carried out in humans show that the intra-uterine environment, especially maternal nutrition, plays an important role in the appearance of metabolic diseases in adulthood [22]. This is the concept of fetal programming, i.e. Developmental Origins of Health and Disease (DOHaD) [10] that must be extended to the first two years of life. The present study adds to the body of literature indicating that studying variation in ^15^N NIA in relation to diet and pre- and early post-natal nutrition might provide decisive information to aid in improved nutrition during a period that determines health in later years.

## Supporting information

S1 Data Data from the study. Sheet 1: F0 data; Sheet 2: F1 data. δ15N: ^15^N NIA (‰); D1: day 1; D11: day 11; D30: day 30; D60: day 60; M2 to M16: month 2 to month 16; D480: day 480; Gest: gestation; Lact: lactation; LPD: low protein diet; NPD: Normal protein diet.
(XLS)

S1 Table ^15^N NIA (‰) values in the liver, muscle and fur over time. Results are Mean±SEM; values with the same letter are not statistically different (multi-way ANOVA and Tukey post-hoc test, significance level, p<0.05).
(DOCX)

S2 Table ^15^N NIA values in different organs and at different time points for NPD/NPD group pooled. Results are Mean±SEM; values with the same letter are not statistically different (multi-way ANOVA and Tukey post-hoc test, significance level, p<0.05).
(DOCX)
