# Supplementary material for: Protein restricted diet during gestation and/or lactation in mice affects 15N natural isotopic abundance of organs in the offspring: Effect of diet 15N content and growth
Source: PLoS One. 2018 Oct 10;13(10):e0205271. doi: 10.1371/journal.pone.0205271 (PMC6179277; doi:10.1371/journal.pone.0205271)
Supplement: S1 Table — Results are Mean±SEM; values with the same letter are not statistically different (multi-way ANOVA and Tukey post-hoc test, significance level, p<0.05). (DOCX) [file pone.0205271.s002.docx]

**S1 Table. ^15^N NIA (‰) values in the liver, muscle and fur over time**

| **Tissue** | **Dietary regime** | **Days** | | | | |
| --- | --- | --- | --- | --- | --- | --- |
|  |  | 1 | 11 | 30 | 60 | 480 |
| **Liver** | NPD/NPD | 7.33±0.10  d | 7.29±0.11  d | 8.38±0.07  a | 8.42±0.08  a | 7.24±0.30  cdef |
|  | LPD/NPD | 6.35±0.08  g | 7.50±0.17  cd | 8.21±0.06  abc | 8.38±0.13  ab | 7.43±0.22  cd |
|  | NPD/LPD | 7.24±0.05  de | 6.32±0.14  fg | 7.30±0.13  de | 8.46±0.18  a | 7.42±0.30  cd |
|  | LPD/LPD | 6.47±0.11  fg | 6.64±0.13  efg | 7.05±0.08  defg | 8.35±0.09  a | 7.49±0.14  bcd |
| **Muscle** | NPD/NPD |  | 7.77±0.09  ac | 7.84±0.06  abc | 7.79±0.08  ac | 7.16±0.21  bcdefg |
|  | LPD/NPD |  | 7.66±0.11  abcd | 7.96±0.13  a | 7.42±0.14  abcdefg | 6.77±0.11  g |
|  | NPD/LPD |  | 7.09±0.11  efg | 7.43±0.06  abcdef | 7.14±0.14  defg | 7.10±0.12  efg |
|  | LPD/LPD |  | 6.88±0.08  fg | 7.17±0.10  bdefg | 7.46±0.11  abcde | 7.12±0.05  defg |
| **Fur** | NPD/NPD |  | 8.58±0.11  a | 8.23±0.22  abc | 7.73±0.30  abcd | 7.90±0.09  abcd |
|  | LPD/NPD |  | 8.42±0.09  ab | 7.71±0.14  bcd | 7.89±0.30  abcde | 6.58±0.32  e |
|  | NPD/LPD |  | 7.72±0.09  bcd | 7.52±0.06  cd | 6.67±0.14  de | 7.44±0.27  cde |
|  | LPD/LPD |  | 7.60±0.14  cd | 7.26±0.07  cde | 6.85±0.22  de | 6.98±0.21  de |

Results are Mean±SEM; values with the same letter are not statistically different (multi-way ANOVA and Tukey post-hoc test, significance level, p<0.05).
