# Supplementary material for: Protein restricted diet during gestation and/or lactation in mice affects 15N natural isotopic abundance of organs in the offspring: Effect of diet 15N content and growth
Source: PLoS One. 2018 Oct 10;13(10):e0205271. doi: 10.1371/journal.pone.0205271 (PMC6179277; doi:10.1371/journal.pone.0205271)
Supplement: S2 Table — Results are Mean±SEM; values with the same letter are not statistically different (multi-way ANOVA and Tukey post-hoc test, significance level, p<0.05). (DOCX) [file pone.0205271.s003.docx]

**S2 Table. ^15^N NIA values in different organs and at different time points for NPD/NPD group pooled**

| **Tissue** | **Days** | | | |
| --- | --- | --- | --- | --- |
|  | 11 | 30 | 60 | 480 |
| **Liver** | 7.29±0.11 e | 8.38±0.07 abc | 8.42±0.08 ab | 7.24±0.30 ef |
| **Muscle** | 7.77±0.09 df | 7.84±0.06 bcdef | 7.79±0.08 cdef | 7.16±0.21 ef |
| **Fur** | 8.58±0.11 a | 8.23±0.22 abcd | 7.73±0.30 bcdef | 7.90±0.09 abcdef |

Results are Mean±SEM; values with the same letter are not statistically different (multi-way ANOVA and Tukey post-hoc test, significance level, p<0.05).
